# Towards an Optimal Energy Consumption for Unattended Mobile Sensor Networks through Autonomous Sensor Redeployment

**DOI:** 10.1155/2014/716838

**Published:** 2014-04-22

**Authors:** Jian Chen, Jie Jia, Yingyou Wen, Dazhe Zhao

**Affiliations:** ^1^Key Laboratory of Medical Image Computing of Ministry of Education, Northeastern University, Shenyang 110819, China; ^2^School of Information Science & Engineering, Northeastern University, Shenyang 110819, China

## Abstract

Energy hole is an inherent problem caused by heavier traffic loads of sensor nodes nearer the sink because of more frequent data transmission, which is strongly dependent on the topology induced by the sensor deployment. In this paper, we propose an autonomous sensor redeployment algorithm to balance energy consumption and mitigate energy hole for unattended mobile sensor networks. First, with the target area divided into several equal width coronas, we present a mathematical problem modeling sensor node layout as well as transmission pattern to maximize network coverage and reduce communication cost. And then, by calculating the optimal node density for each corona to avoid energy hole, a fully distributed movement algorithm is proposed, which can achieve an optimal distribution quickly only by pushing or pulling its one-hop neighbors. The simulation results demonstrate that our algorithm achieves a much smaller average moving distance and a much longer network lifetime than existing algorithms and can eliminate the energy hole problem effectively.

## 1. Introduction

A typical wireless sensor network (WSN) is composed of hundreds of sensor nodes reporting their data to the information collector, referred to as the sink node. Sensor nodes are usually of low cost and low power, having limited sensing, computing, and communication capabilities. In recent years, with the rapid progress in advanced VLSI and radio frequency (RF) technologies, WSNs have attracted lots of interest due to their potential use in various applications such as military surveillance, target tracking, emergency navigation, and large scale systems [1, 2].

Sensor deployment is an important issue in designing a WSN since it affects the communication cost, detection capability, coverage, and connectivity [3]. In general, the resource-limited sensor nodes are dropped from airborne vehicles for remote surveying of unattended environment without a preconfigured infrastructure. Those sensor nodes are typically left unattended and remain static after initial deployment. Sometimes, establishing such a fixed sensor network over a hostile or dangerous area to provide complete coverage could be a daunting task. Thus, there exists an urgent need for exploiting sensor mobility in WSNs to improve network performance.

As sensor nodes are usually battery driven, they can survive for only a limited lifetime with nonrenewable batteries. Taking it one step further, the limited constraints of the sensor nodes restrict the use of high complexity algorithms and protocols. How to balance energy consumption is one of the fundamental issues arising in WSN. To address this issue, much work has been done during recent years. Among them, taking advantage of sensor mobility to enhance network lifetime has attracted extensive attention [4–11]. Typically, most of these traditional approaches aimed at having uniform deployment to achieve full coverage using a minimum number of sensor nodes. However, these approaches do not consider the issue of unbalanced energy depletion with distance to a predetermined sink. As the source nodes sent their sensing messages to the sink via a multihop relay tree in WSN, this many-to-one communication pattern could lead to traffic imbalance among sensor nodes. It has been observed that the closer a sensor is to the sink, the faster its battery exhausts, as time evolves, which will cause an energy hole in the region near the sink [12]. If this happens, no more data could be transmitted to the sink and the network is inactive soon leaving much energy unused, regardless of how many sensor nodes are deployed. Experimental results in [13] show that when the network lifetime ends, nearly 90% of the total initial energy is unused with uniform distribution. Therefore, managing sensor mobility to achieve both optimal energy consumption and full coverage is important to prolong the network lifetime for WSN.

In this paper, we try to solve the energy hole problem by proposing an autonomous coverage-driven sensor redeployment scheme. We first develop the energy hole problem with nonuniform node distribution in WSN theoretically. By importing the energy-aware transmission mechanism and the accessibility condition of energy-balanced depletion in our pervious approach [14], we further propose a fully distributed density control scheme in different coronas to balance energy depletion for the entire network. The rest of the paper is organized as follows.  Section 2 reviews the related literature.  Section 3 describes the network model, assumptions, and energy-aware transmission mechanism. A novel sensor redeployment strategy is proposed in Section 4.  Section 5 presents simulation results for our algorithms, and Section 6 concludes our paper.

## 2. Related Work 

More recently, there has been growing interest in optimizing the sensor movement to maintain full coverage and prolong the network lifetime for mobile sensor networks. In [6], the authors proposed a potential field-based deployment algorithm, in which all the nodes explore from a compact region and fill the maximum working area in a way similar to the particles in the microworld [15]. In [7], the authors assumed that there were virtually attractive and repulsive forces among sensors. Using these virtual forces, mobile sensor nodes spread throughout the target area with a uniform distribution such that the network coverage rate is maximized. In [8], the authors proposed a Voronoi diagram-based distribution model, in which each sensor iteratively calculates its Voronoi polygon to detect the coverage holes and moves to a better position to improve the coverage rate. In [9], the authors proposed three independent algorithms (VEC, VOR, and MiniMax), by pushing or pulling nodes to cover the gaps based on virtual forces. These three algorithms have comparable performance in a bounded area, whereas only VEC algorithm can be used in both unbounded and bounded areas. In [10], the authors investigate how to move sensors to some locations while still preserving the degree of coverage under partially controlled placement. In [11], the sensing field was split into grids, and the sensors moved from high-density grids to low-density ones such that the densities remain constant. Overall, most of these traditional algorithms intended to redistribute sensor nodes uniformly and thus to maximize coverage rate, minimize coverage overlap or gap, and reduce the network cost for WSN. However, as the uniform distribution has unbalanced communication traffic, these approaches will cause the network lifetime to end prematurely with a great amount of energy unused.

In [12], the authors investigated the problem of uneven energy consumption in many-to-one WSNs for the first time. Further, they proposed several approaches to mitigate this problem and inferred that simply increasing the number of nodes under a uniform distribution could not prolong the network lifetime [16]. In [17], the authors focused on the nonuniform energy distribution among sensors. It was concluded that the higher the workload was, the higher the initial level of energy would be set. Although such a strategy seems to be promising, its application may be so difficult that it is inconvenient in producing and deploying sensor nodes. Sink mobility was also introduced to tackle the energy hole problem [18–20]. The authors in [18] proposed an autonomous sink movement strategy in which mobile sinks move toward half-quadrant zones with abundant energy. Similarly, the authors in [19] drew a conclusion that utilizing mobile sink could prolong network lifetime 3.48 times at most compared to the case with static sinks (when the mobile sink moves around a hexagonal network perimeter and stops at the six corners). They also proposed a sink movement algorithm to keep network connectivity so as to prolong network lifetime. The authors in [20] tried to incorporate both static and mobile sinks to improve the network performance of WSN, in which the static sinks are located at the center of the target area and the mobile sink moves around the network perimeter. Each time when the mobile sink stops at a new location, it only needs to broadcast the location updates messages to a subset of sensor nodes. Despite the fact that mobile sinks would bring some advantages to WSN, some new issues appeared such as the movement control policy, the energy-aware routing protocols, and the efficient path planning. Unfortunately, these issues are very complicated and not readily solvable [21, 22].

Energy-aware sensor redistribution was also proposed to mitigate or avoid the energy hole problem in WSNs. In [23], the authors proposed an efficient node placement, topology control, and a scheduling protocol of the MAC layer to prolong the network lifetime for a grid-based WSN. In [24], the authors developed a mixed-integer linear programming model for determining the locations of sensors and sinks, schedules, and sensor-to-sink data flow routes. Further, they proposed a heuristic algorithm to maximize the network lifetime. In [25], the authors tried to solve the energy provisioning and relay node placement problems simultaneously in a two-tiered WSN. At first, the low-cost sensors sense the surrounding area and forward the sensing data to their cluster head, and then the cluster head forwards the data to the sink. Further, they proposed a heuristic approach to solve the mixed-integer nonlinear programming problem. In [26], the authors investigated sensor self-deployment problem, by constructing focused coverage around a point of interest. The authors in [27] explored variable node distribution density to mitigate the effects of the uneven energy depletion. The authors in [28] proposed a nonuniform distribution algorithm to solve the energy hole problem in a corona-based WSN. With their theoretical analysis, when all the sensors have a constant data acquisition rate, the balanced energy depletion among the whole network is impossible. However, their traffic pattern may not be true for highly dense WSN and the uneven energy depletion still exists between the outermost corona and the inner coronas. In fact, we can prove that balanced energy depletion is achievable with the extra help of energy-aware transmission mechanism in this paper. In [29], the authors investigated the sink-hole problem in duty-cycled connected *k*-covered WSNs, where each point is covered by at least *k* sensor. In [30], the ring-based relays are also used to eliminate energy hole of a WSN; however, how to form such a relay is not discussed in their approach. In our pervious approach [14], we have proposed the centralized algorithm to form an energy-balanced distribution for WSN; however, the exchange of global location information during dynamic sensor movement would put a heavy traffic burden on the network. In [31], the authors proposed a transmission range adjustment approach to tackle the unbalanced energy depletion. However, searching for the optimal transmission ranges among all the coronas is a NP-complete problem.

Overall, though most of the algorithms discussed above intended to maximize coverage rate, minimize deployment density, and eliminate the energy hole, they did not answer a fundamental question in sensor redeployment: what type of node layout and communication pattern that could provide the maximum coverage with the smallest overlap and gap and guarantee that all the working sensors die simultaneously with nearly zero residual energy? We will deal with this issue in the next section.

## 3. Network Model and Assumptions

In this section, we will present our network model and basic assumptions. Assume that a set of *N* homogeneous sensors with the same initial energy *ε* is deployed in a circular area with radius *d* to monitor some physical phenomena and an unlimited amount of energy is set for the sink node. We refer to the set of deployed sensors as *S* = {*s*_1_, *s*_2_,…, *s*_*N*_}, each of which has an ID, a fixed transmission range *R*_*c*_, a fixed sensing range *R*_*s*_, and is aware of its location. The only sink node is placed at the center of the circle. We divide the area into *n* adjacent coronas with the equivalent width of *R*_*c*_, and the *i*th corona is denoted by *C*_*i*_. Obviously, the corona *C*_*i*_ is composed of nodes whose distances to the sink node are between (*i* − 1)∗*R*_*c*_ and *i*∗*R*_*c*_.

In this paper, periodic data gathering monitoring is considered, where the network is working in rounds. Each round is further divided into two phases: the node redistribution phase and stability monitoring phase. We will provide a detailed description of the first stage issues in the next section. During the second phase, each working node should send their sensing messages to the sink node per unit time via multihop communication. For the theoretical analysis, we use a simplified power consumption model and an ideal MAC layer with no collisions and retransmissions. The initial energy of each sensor is set as *ε* > 0, and the sink has no energy limitation. We further assume that each sensor consumes *e*_1_ units of energy when sending one bit, while it depletes *e*_2_ units of energy when receiving one bit, where *e*_1_ > *e*_2_ > 0.

When using traditional transmission mechanism, the redundant sensing messages for the same area will be retransmitted by more than one sensor, consuming a considerable amount of energy [26]. In order to save energy, an energy aware data transmission mechanism proposed in our pervious approach [14] is imported. In this mechanism, we first need to build Voronoi graph for each sensor node to determine its own sensing range. After the establishment of the Voronoi graph, the sensing message for each pixel is sent only once. Based on the network model, we can conclude that sensors belonging to corona {*C*_*i*_ | *i* ≠ *n*} will forward both the data generated by themselves and the data produced by coronas {*C*_*j*_ | (*i* + 1) ≤ *j* ≤ *n*}. Obviously, the sensors located in the outermost corona *C*_*n*_ do not need to forward any data. Assume that the sensors in each corona are distributed uniformly and there is no data aggregation at any forwarding nodes. Define the number of sensors deployed in corona *C*_*i*_ as *N*_*i*_ and the number of pixels in *C*_*i*_ as *A*_*i*_. When the energy-aware data transmission mechanism is applied, the number of messages for corona *C*_*i*_ to receive and forward is (*A*_*i*+1_ + *A*_*i*+2_ + ⋯+*A*_*n*_) and (*A*_*i*_ + *A*_*i*+1_ + ⋯+*A*_*n*_). As sensing messages are transmitted per working round, the average energy consumption per working round of sensors in corona *C*_*i*_ is

(1) $$\begin{array}{c} {\overset{-}{E}}_{i}=\frac{e_{1}}{\rho_{i}}+\frac{A_{i+1}+A_{i+2}+\cdots +A_{n}}{A_{i}\cdot \rho_{i}}\left(e_{1}+e_{2}\right),\;1\leq i\leq n-1, \end{array}$$

where *ρ*_*i*_ is the node density of corona *C*_*i*_.

As the sensors in corona *C*_*n*_ only need to send their own sensing messages, the energy depletion of sensors in corona *C*_*n*_ can be calculated as follows:

(2) $$\begin{array}{c} {\overset{-}{E}}_{n}=\frac{A_{n}\cdot e_{1}}{N_{n}}=\frac{e_{1}}{\rho_{n}}. \end{array}$$

**Definition 1**

The optimal energy consumption state means that all the sensors in the network deplete their energy in the constant ratio; namely, all the sensor nodes have the same lifetime which is identical with the corresponding network lifetime. In particular, if the optimal energy depletion is achieved, there is no energy wasted and the network lifetime can be given by

(3) $$\begin{array}{c} \frac{\varepsilon }{{\overset{-}{E}}_{1}}=\frac{\varepsilon }{{\overset{-}{E}}_{2}}=\cdots =\frac{\varepsilon }{{\overset{-}{E}}_{i}}=\cdots =\frac{\varepsilon }{{\overset{-}{E}}_{n}}. \end{array}$$

**Theorem 2**

Optimal energy depletion is possible, if all the working nodes take the energy-aware transmission mechanism, and the node density *ρ*_*i*_ in corona *C*_*i*_ satisfies [14]

(4) $$\begin{array}{c} \rho_{i}=\rho_{n}\cdot \left[1+\frac{\left(n^{2}-i^{2}\right)\cdot \left(e_{1}+e_{2}\right)}{\left(2i-1\right)\cdot e_{1}}\right],\;\rho_{1}\geq \rho_{2}\geq \cdots \geq \rho_{n}. \end{array}$$

**Proof**

Without loss of generality, suppose that (4) is true: so (1) can be described as follows:

(5) E−i=Ai·e1+∑k=i+1nAk·(e1+e2)Ai·ρi=[Ai·e1+∑k=i+1nAk·(e1+e2)]·(2i−1)·e1Ai·ρn·[(2i−1)·e1+(n2−i2)·(e1+e2)].

As *A*_*i*_ = *πR*_*c*_^2^ · (2*i* − 1), we have

(6) E−i=e1ρn=E−n.

Since *dρ*_*i*_/*di* = (−2*i*/(2*i* − 1)) − (2(*n* ^2^ − *i* ^2^)/(2*i*−1)^2^) < 0 is a permanent establishment, we can draw a conclusion that *ρ*_1_ ≥ *ρ*_2_ ≥ ⋯≥*ρ*_*n*_. This completes the proof of Theorem 2.

Theorem 2 shows that, in a circular monitored area, based on the energy-aware transmission mechanism, if the sensors in each corona obey a uniform distribution and the node density meets a certain condition, the optimal energy depletion of the whole network can be achieved. Besides, the node density *ρ*_*i*_ of corona *C*_*i*_ only depends on *ρ*_*n*_ and the corona number *i*.

**Theorem 3**

The lifetime comparison of energy–balanced redeployment with traditional uniform approaches is *ρ*_1_/*ρ*_*N*_.

**Proof**

Suppose that these two schemes run with the same initial conditions. The node density of each corona obeys (4) in our proposed scheme, while, in the uniform distribution, the density *ρ*_*i*_ is equal to *ρ*_*n*_. As the innermost corona *C*_1_ needs to forward all the sensing messages in the whole network, it consumes the most energy. Therefore, the maximum network lifetime under uniform distribution is determined by the survival time *C*_1_. The network lifetime can be calculated as

(7) εE−1′=ρn·εe1+(n2−1)·(e1+e2),

where ${\overset{-}{E}}_{1}^{\prime }$ is the average energy depletion of *C*_1_ per unit time under uniform distribution. Further, we can get the average energy depletion in *C*_1_ under optimal energy consumption conditions as

(8) $$\begin{array}{c} {\overset{-}{E}}_{1}=\cdots {\overset{-}{E}}_{i}=\cdots ={\overset{-}{E}}_{n}=\frac{e_{1}}{\rho_{n}}. \end{array}$$

Thus the lifetime enhancement is

(9) $$\begin{array}{c} \frac{\varepsilon /{\overset{-}{E}}_{1}}{\varepsilon /{\overset{-}{E}}_{1}^{\prime }}=\frac{\left(e_{1}+\left(n^{2}-1\right)\cdot \left(e_{1}+e_{2}\right)\right)/\rho_{n}}{e_{1}/\rho_{n}}=\frac{\rho_{1}}{\rho_{n}}>1. \end{array}$$

This completes the proof of Theorem 3.

Therefore, compared with traditional uniform distribution strategy, the network lifetime can raise as much as *ρ*_1_/*ρ*_*n*_ times by using optimal energy depletion distribution.

## 4. Optimization of Sensor Redeployment 

In this section, we first introduce equivalent sensing radius. Then we develop the uniform sensor distribution for corona-shaped area. Further, we propose a novel autonomous sensor redeployment approach to balance energy depletion.

### 4.1. Redistribution under Equivalent Sensing Radius

**Definition 4**

Equivalent sensing radius is defined as the sensing radius when the given distribution density *ρ*_*i*_ is the lowest one to maintain the full coverage for the target area with size *S*.

According to [9], the equivalent sensing radius and the distribution density *ρ*_*i*_ satisfy the following:

(10) $$\begin{array}{c} R_{i}=\sqrt{\frac{2}{\sqrt{27}\cdot \rho_{i}}}. \end{array}$$

**Theorem 5**

Optimal energy depletion can be achieved, where *R*_*i*_ satisfies the following:

(11) Ri=Rs(2i−1)·e1(2i−1)·e1+(n2−i2)·(e1+e2),            R1≤R2≤⋯≤Rn.

**Proof**

According to the definition of equivalent sensing radius, we can combine it with the energy balance condition. Thus we have

(12) ρi=227·Rs2·[1+(n2−i2)·(e1+e2)(2i−1)·e1].

After simple transformation, we can get

(13) $$\begin{array}{c} R_{i}=R_{s}\cdot \sqrt{\frac{\left(2i-1\right)\cdot e_{1}}{\left(2i-1\right)\cdot e_{1}+\left(n^{2}-i^{2}\right)\cdot \left(e_{1}+e_{2}\right)}}. \end{array}$$

This concludes the proof of Theorem 5.

Since the equivalent sensing radius is only determined by corona number *i*, the redeployment of sensors in corona *C*_*i*_ is similar to the traditional uniform distribution problem. Define the equivalent sensing radius of corona *C*_*i*_ as *R*_*i*_ and the desired number of deployed sensor for *C*_*i*_ as *ρ*_*i*_ · *S*_*i*_. Note that the boundary effects cannot be ignored simply. In order to cover corona *C*_*i*_ uniformly, the optimal sensor distribution should satisfy the following conditions.

(1) If *R*_*i*_ ≥ *R*_*c*_/2, the optimal deployment can be achieved if all the sensors are uniformly lying on the middle line of corona *C*_*i*_, and the angle between any two adjacent nodes is *α*_*C*_*i*__ = 2*π*/(*ρ*_*i*_ · *S*_*i*_).
(2) If *R*_*i*_ < *R*_*c*_/2, as the single ring distribution will cause coverage gaps, the optimal sensor redeployment is equivalent to a multiring distribution. Define the number of rings for *C*_*i*_ as *N*_*i*_, calculated as *N*_*i*_ = ⌈*R*_*c*_/2*R*_*i*_⌉. When multiring uniform distribution is achieved for corona *C*_*i*_, the number of sensors distributed in *k*_th_ ring *C*_*i*_^*k*^ is *ρ*_*i*_ · *S*_*C*_*i*_^*k*^_, and the angle between any two neighbours in *C*_*i*_^*k*^ is *α*_*C*_*i*_^*k*^_ = 2*π*/(*ρ*_*i*_ · *S*_*C*_*i*_^*k*^_), where *S*_*C*_*i*_^*k*^_ is the area of ring *C*_*i*_^*k*^. Assuming that all the rings have the same width, *d* = *R*_*c*_/*N*_*i*_, such that *S*_*C*_*i*_^*k*^_ can be calculated as
  (14) SCik=π[(i−1)·Rc+k·d]2 −π[(i−1)·Rc+(k−1)·d]2.

**Figure 1:**
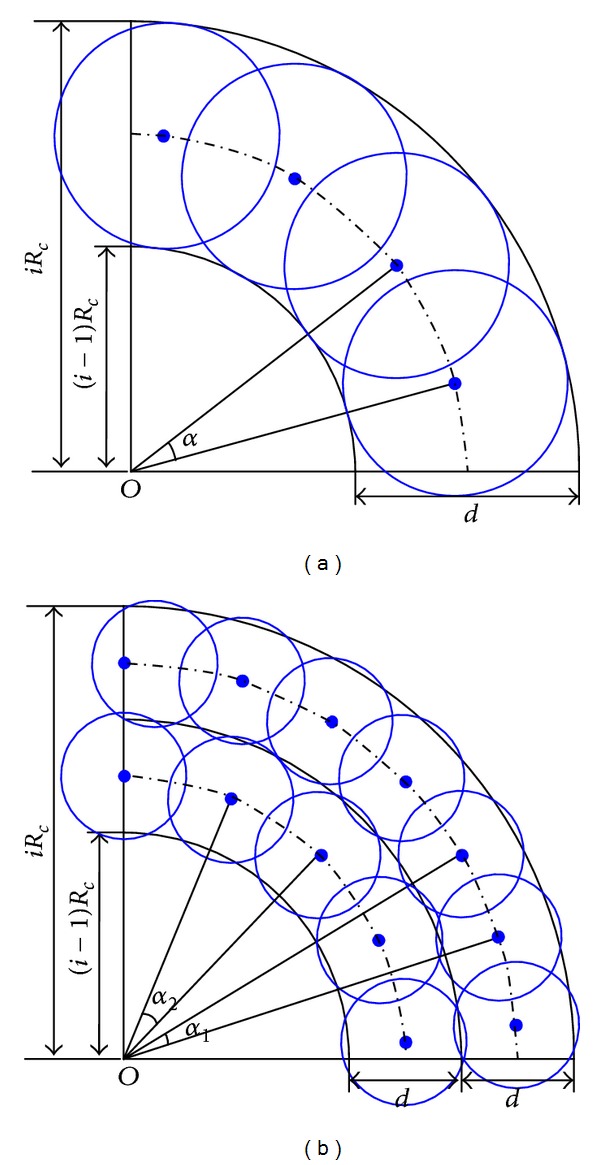
Optimal sensor redeployment with different equivalent sensing radii *R*_*i*_. (a) *R*_*i*_ = *R*_*c*_/2: uniform distribution in single ring; (b) ⌈*R*_*c*_/2*R*_*i*_⌉ = 2: uniform sensor distribution in two rings.

Figure 1 shows the optimal sensor redeployment with different equivalent sensing radii *R*_*i*_. When *R*_*i*_ = *R*_*c*_/2, the sensor nodes in *C*_*i*_ obey a single-ring uniform distribution, and the angle between any two neighbours is *α* = 2*π*/(*ρ*_*i*_ · *S*_*i*_), as shown in Figure 1(a). When ⌈*R*_*c*_/2*R*_*i*_⌉ = 2, the sensor nodes in corona *C*_*i*_ obey a two-ring uniform distribution; the adjacent angles of ring *C*_*i*_^1^ and *C*_*i*_^2^ are *α*_*C*_*i*_^1^_ = 2*π*/(*ρ*_*i*_ · *S*_*C*_*i*_^1^_) and *α*_*C*_*i*_^2^_ = 2*π*/(*ρ*_*i*_ · *S*_*C*_*i*_^2^_), respectively.

Therefore, the optimal sensor redeployment to balance energy depletion can be transformed into a uniform distribution problem with given deployment density and equivalent sensing radius. The novel sensor redeployment algorithm mainly contains two parts: (1) sensor redeployment control among the coronas to regulate the number of nodes for each corona and (2) sensor redeployment control inside coronas to guarantee that each corona achieves the given node density.

### 4.2. Distributed Sensor Redeployment among Coronas

As sensors are randomly deployed in the target area, the deployment uncertainty may cause the number of deployed nodes to be more or fewer than the corona really needs. Movement control of sensor nodes will satisfy the desired deployment density for each corona and its rings. To avoid consuming much energy during the moving process, the nodes are only allowed to move to the adjacent coronas. After the needed number of sensors is achieved in each corona, a certain number of sensors should move between different rings to achieve uniform distribution. Define the number of sensors deployed in corona *C*_*i*_ as *D*_*i*_ and the desired number of sensors in *C*_*i*_ as *ED*_*i*_. Sensor redeployment control among coronas can be conducted in Algorithm 1.

### 4.3. Distributed Sensor Redeployment inside Coronas

**Figure 2:**
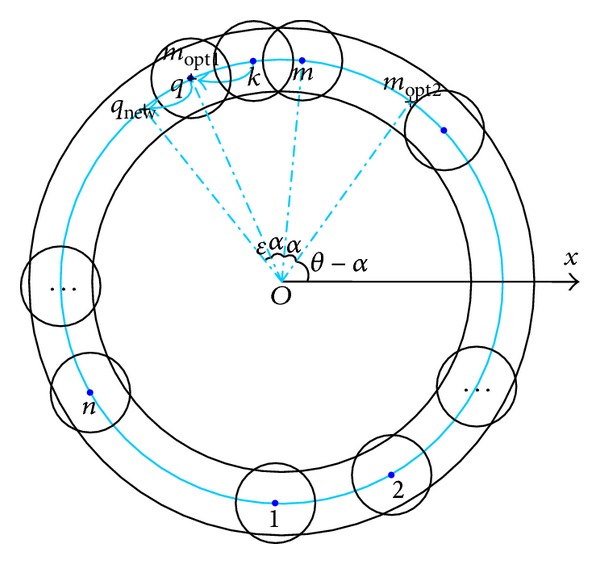
Sensor redeployment in the ring.

Sensor redeployment inside coronas mainly focuses on how to redistribute the nodes locating on the median line of these rings in each corona to a perfect layout. Here, it is executed simply with its neighbour nodes. The straightforward idea is to adjust the angle between any two neighbours to *α*. However, as each node may have numerous neighbours, the nearest neighbour is taken in the current working round. Taking Figure 2 as an example, assume that node *m* is the nearest neighbour of node *k*, to form an optimal layout and node *k* should move to *m*_opt1_ or *m*_opt2_, both of which are the best locations of node *m*. In our algorithm, node *k* moves straight to the nearer location *m*_opt1_, thus saving energy. If some other sensors *q* existed in this best location, to minimize the total movement among all the sensors, *q* should move with a small deviation *ε* along the same orientation to node *m*. The whole process of sensor redeployment inside coronas is shown in Algorithm 2.

**Theorem 6**

The sensor redeployment algorithm ends in finite rounds and can achieve uniform layout in the ring.

**Proof**

As long as the angle between any two nearest neighbours is not equal to *α*, the total covered area with uniform distribution will keep on growing. It suffices to prove that, once the algorithm has reached the stable state when the angle between any two neighbours is equal to *α*, the sensors locating on the ring will be fixed.

Let *S* = (*x*_1_, *x*_2_,…, *x*_*n*_) denote the set of sensors distributed in the ring, whereas *θ*_*i*_ ∈ (0, 2*π*] is azimuth between the lines *ox*_*i*_ and *ox*. After reordering these sensors according to *θ*_*i*_, we rename *S* as (*θ*_1_, *θ*_2_,…, *θ*_*n*_). Define *β*_*i*_ as the angle of two adjacent nodes in *S*. To ensure that *β*_*i*_ does not exceed *α*, it needs to satisfy the following:

(15) $$\begin{array}{c} \beta_{i}=\{\begin{array}{cc} \theta_{i+1}-\theta_{i} & \text{if}\left(\theta_{i+1}-\theta_{i}\right)\leq \alpha \\ 0 & \left(\theta_{i+1}-\theta_{i}\right)>\alpha . \end{array} \end{array}$$

Thus the total covered angle of *S* can be calculated as

(16) $$\begin{array}{c} f\left(S\right)=\overset{n-1}{\underset{i=1}{\sum }}\beta_{i}. \end{array}$$

If all the sensor nodes are distributed in an optimal layout, we can get the upper bound for *f*(*S*) as 2*π* − *α*.

Denote the angle between *θ*_*i*_ and *θ*_*i*+1_ as *β*_*i*_. We now consider the changes of *β*_*i*_ in one working round. If *β*_*i*_ < *α*, the following three cases may occur.

**Figure 3:**
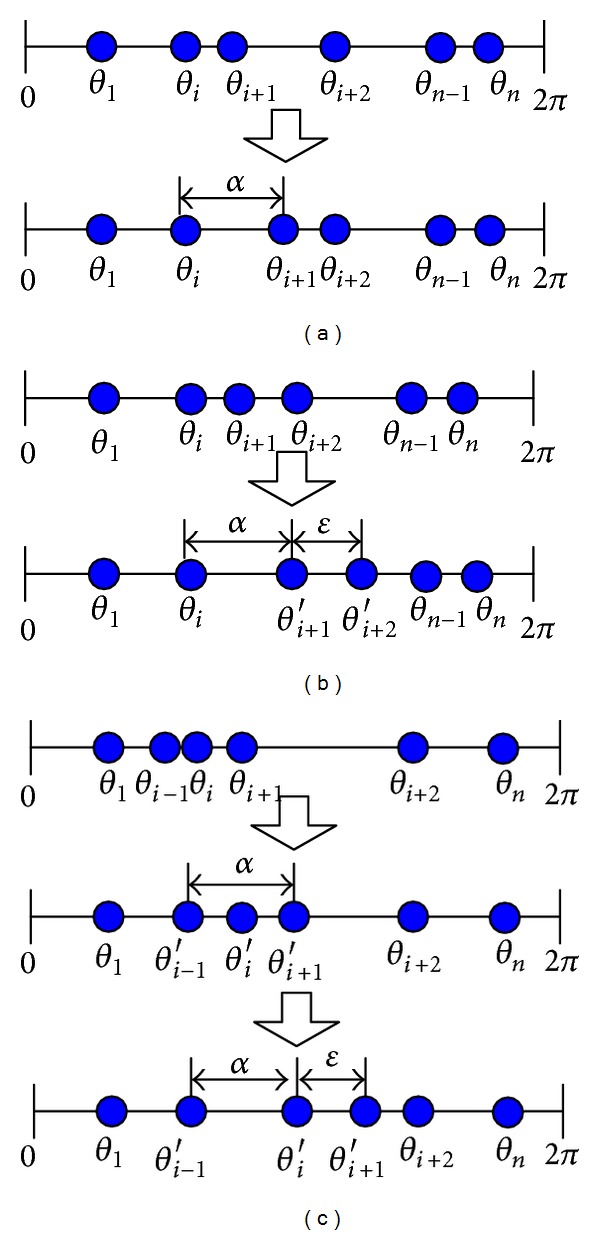
Illustration of sensor redeployment in one working round.

(1) If *β*_*i*_ increases to *α* with no sensors coinciding, the new achieved distribution can be expressed as *S*′ = (*θ*_1_, *θ*_2_,…, *θ*_*i*_, *θ*_*i*+1_′,…, *θ*_*n*_), as shown in Figure 3(a). Since *β*_*i*_′ > *β*_*i*_, we have *f*(*S*′) > *f*(*S*).
(2)  If *β*_*i*_ increases to *α* with some sensors coinciding, the coincided sensors would move with a small deviation *ε* in our algorithm. Assume that the new achieved distribution is *S*′ = (*θ*_1_, *θ*_2_,…, *θ*_*i*_, *θ*_*i*+1_′, *θ*_*i*+2_′,…, *θ*_*n*_), as shown in Figure 3(b). Therefore, *f*(*S*′) can be calculated as
  (17) f(S′)=∑j=1i−1βj+(θi+1′−θi)+(θi+2′−θi+1′) +∑j=i+2nβj=∑j=1i−1βj+α+ε+∑j=i+2nβj,
  while
  (18) f(S)=∑j=1i−1βj+(θi+1−θi)+(θi+2−θi+1) +∑j=i+2nβj=∑j=1i−1βj+α+∑j=i+2nβj.
 Thus we have *f*(*S*′) > *f*(*S*).
(3) If *β*_*i*_ increases to *α* with some sensors crossing, the new achieved distribution can be denoted as *S*′ = (*θ*_1_, *θ*_2_,…, *θ*_*i*−1_′, *θ*_*i*_′, *θ*_*i*+1_′,…, *θ*_*n*_), as shown in Figure 3(c). Since this new generated distribution is similar to case (2), we can draw the same conclusion of *f*(*S*′) > *f*(*S*), when *θ*_*i*_′ becomes the next operating node.

Clearly, in all the cases, *f*(*S*) is an increasing function on rounds (1,…, *n*). After a finite number of rounds, *f*(*S*) would achieve its upper bound. As a result, all the sensors will be redeployed uniformly on the middle line of the ring with the given density. This completes the proof of Theorem 6.

## 5. Performance Evaluation

In this section, we will present the simulation results of our algorithm for both random and Gaussian deployment models. Three metrics, including coverage rate, the average moving distance, and the network survival lifetime, are imported to evaluate the performance of the algorithm.

First, a random deployment is considered in the circular area of radius 100 with 627 potential sensors. We assume that all the sensors are homogeneous, that is, having the same initial energy reserve *ε* of 10000 J, the same transmission radius *R*_*c*_ of 25 m, and the same sensing radius *R*_*s*_ of 9 m, respectively. The values of *e*_1_ and *e*_2_ are given as follows: *e*_1_ = 0.5/10^3^ J/bit and *e*_2_ = 0.25/10^3^ J/bit. The total number of working rounds for sensor node *i* is determined by *ε*/(*L* · *M*_*i*_ · *e*_1_ + *L* · *N*_*i*_ · (*e*_1_ + *e*_2_)), where *M*_*i*_ is the total number of pixels covered by *i*, *N*_*i*_ is the total number of messages forwarded by *i*, and *L* is the length of each sensing pixel, *L* = 1000 bits.

The sensing data forwarding strategy is similar to [27]. As the nodes obey an approximate uniform distribution in the corona after sensor redeployment, any node in corona *C*_*i*_ can directly communicate with almost *ρ*_*i*−1_ · *A*_*i*−1_/*ρ*_*i*_ · *A*_*i*_ nodes in *C*_*i*−1_. Among these *ρ*_*i*−1_ · *A*_*i*−1_/*ρ*_*i*_ · *A*_*i*_ candidate nodes, the node with most residual energy will be selected as the forwarding one.

**Figure 4:**
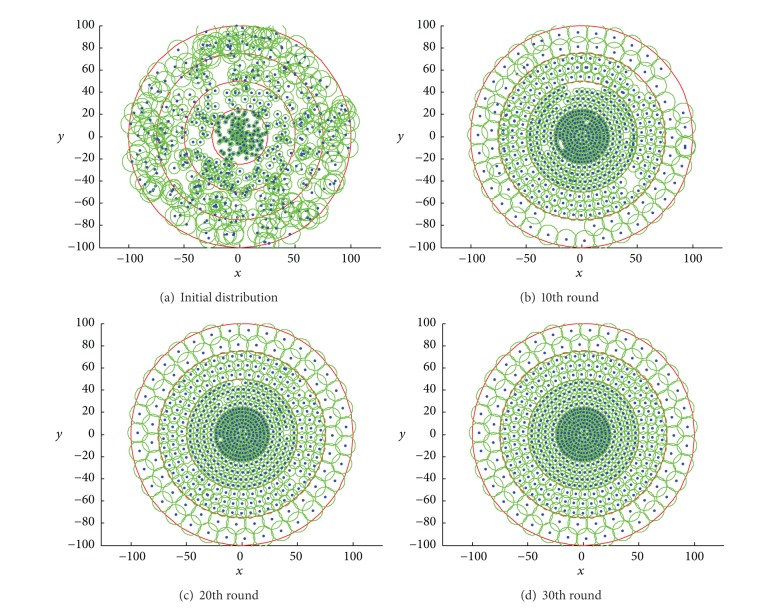
Illustration of network distribution in different rounds using random deployment model.

Figure 4 depicts the sensor distribution gained in different rounds using random deployment model. The target area is divided into four coronas marked from *C*_1_ to *C*_4_, and the equivalent sensing radius from *C*_1_ to *C*_4_ can be calculated as 1.86, 3.4, 5.11, and 9, respectively, as shown in Figure 4(a). From the simulation results, we can make the observation that our proposed algorithm can converge to the global optimal distribution effectively. What is more, the layout shown in Figure 4(d) is the optimal uniform sensor distribution with the given density, which achieves the coverage rate of 97.6%. And the number of sensors redeployed from corona *C*_1_ to *C*_4_ is 220, 196, 145, and 66, which approaches to the accessibility condition of optimal energy depletion.

The second assumption examined is the Gaussian distribution, and the simulation environment is the same as the first experiment. In this distribution, each sensor's coordinate (*x*, *y*) obeys a two-dimensional normal Gaussian function:

(19) $$\begin{array}{c} f\left(x,y\right)=\frac{1}{2\pi \sigma^{2}}e^{-[x^{2}+y^{2}]/2\sigma^{2}}, \end{array}$$

where *σ* represents the standard deviation of Gaussian distribution. We set *σ* equal to the communication radius *R*_*c*_ to ensure that all the sensors will be deployed in the target area.

**Figure 5:**
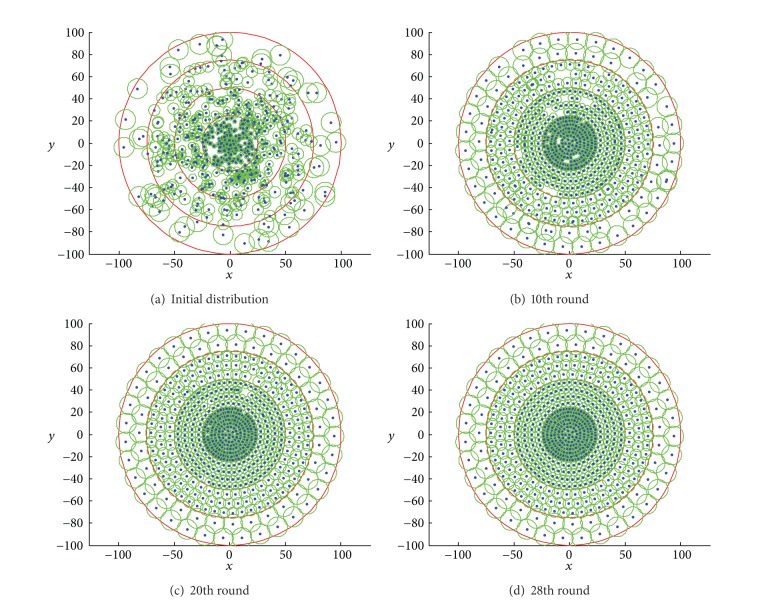
Illustration of network distribution in different rounds using Gaussian deployment model.

Figure 5(a) shows the sensor distribution obtained in different rounds using Gaussian deployment model. From the simulation results, we can draw a conclusion that the total coverage enlarges round by round, as observed from Figure 5(b) to Figure 5(d). At the 28th round, the nodes are uniformly distributed with no sensors moving anymore, which indicates that the algorithm can converge to the optimal solution quickly.

**Figure 6:**
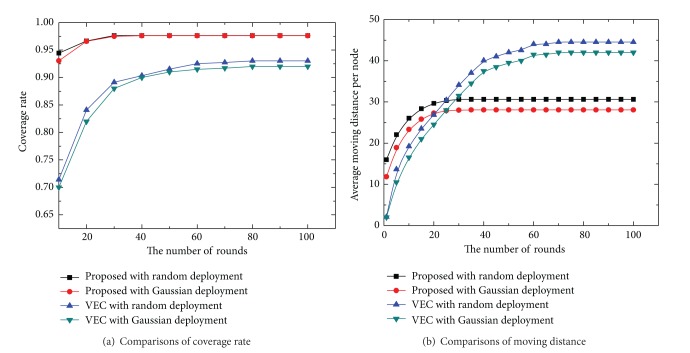
Comparisons of network performance in different rounds.

Further, we compare the performance of our algorithm with VEC in terms of coverage rate and average moving.  Figure 6 shows the average moving distance and coverage rate for VEC [10] and our approach in different numbers of rounds. From the simulation results in Figure 6, we can conclude that (1) the average moving distance under Gaussian deployment model is shorter than under random model, which is mainly due to having more sensors deployed in the central region under Gaussian deployment model; (2) to achieve the desired coverage rate, many more sensors are needed in VEC than in our proposed algorithm, which shows our algorithm has a shorter average moving distance than VEC under the same condition; (3) our algorithm can get a higher coverage rate than VEC, which means that our algorithm is free of the boundary influence and can achieve the optimal sensor distribution effectively.

We further compare the performance of our algorithm with traditional uniform and nonuniform sensor deployment in terms of network lifetime and energy efficiency. In addition, the performance of our energy-aware transmission mechanism (*E*_*t*_) and traditional transmission mechanism (*T*_*t*_) combined these algorithms is also tested.

Since the node densities for all the coronas are the same in uniform deployment, the number of sensors distributed from *C*_4_ to *C*_1_ is 274, 196, 118, and 39. While fewer nodes are used to cover the outmost corona in the nonuniform deployment, the ratio *q* is set to 2.12. Therefore, the number of sensors uniformly distributed from corona *C*_4_ to *C*_1_ is 66, 73, 156, and 332, respectively. For our algorithm, to ensure the optimal energy consumption, the number of sensors distributed from corona *C*_4_ to *C*_1_ is 66, 145, 196, and 220.

**Figure 7:**
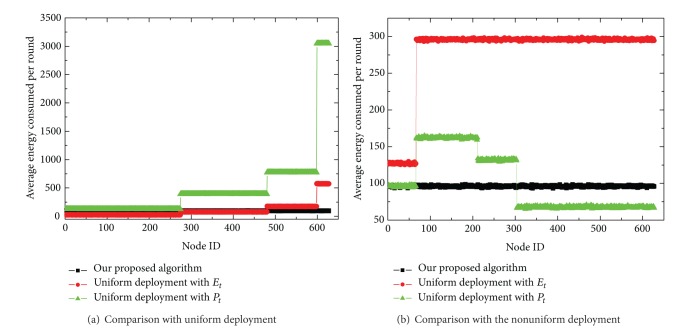
Comparison of average energy consumption in a sampling period.

Figure 7 shows the comparisons of average energy depletion per working round. From the simulation results in Figure 7, we can draw the conclusions that (1) the average energy consumed in a round in *E*_*t*_ is much smaller than in *T*_*t*_. Because the energy-aware transmission can avoid retransmitting the same sensing data, the total messages transferred in each round are much smaller; (2) since the uniform deployment ignores the traffic imbalance, the innermost corona has the largest traffic load and most energy consumption, as shown in Figure 7(a); (3) the nonuniform deployment combined with *P*_*t*_ scheme can achieve a suboptimal distribution; (4) the energy consumption of the whole working set is almost equal in our approach. Although the nodes in inner coronas do not behave as source but router, their sensing pixels are much smaller than those of the outer coronas.

**Figure 8:**
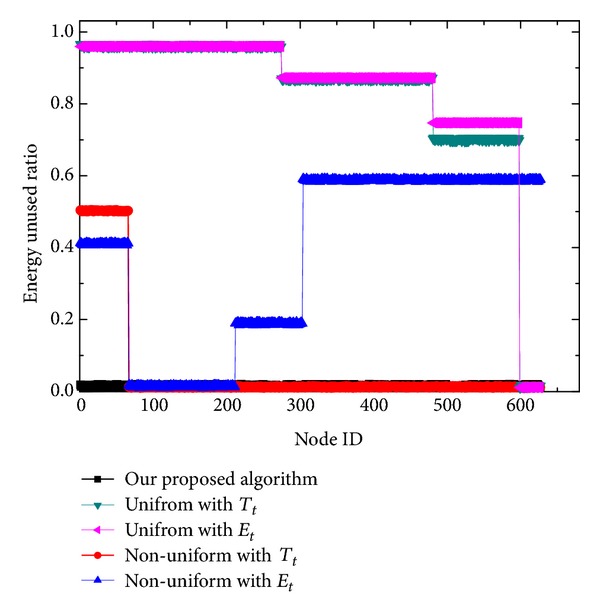
Comparison of energy unused ratio for each node.

Figure 8 compares the energy unused ratio of each node when the network terminates. Here, the energy unused ratio refers to the ratio of the residual energy to the initial energy at the end of the network lifetime. From Figure 8, clearly, the uniform scheme leaves an abundant amount of nodal energy in both transmission mechanisms as expected, especially for the sensor nodes in the outermost corona. Although the nonuniform deployment combined with *T*_*t*_ scheme achieves much better energy efficiency, it leaves a considerable amount of unused energy in the outermost corona (almost 50% or so of the initial nodal energy). For the nonuniform deployment combined with *P*_*t*_, as corona *C*_3_ has the most transmission load and corona *C*_1_ has the largest node density, no doubt, *C*_1_ has the largest energy unused ratio. For our algorithm, as the energy consumption of the whole working set is almost equal, all the sensors exhaust their energy simultaneously when the network terminates, as shown in Figure 7. In fact, the energy unused ratio for most sensors is lower than 1%.

Table 1 compares these algorithms in network lifetime. We can see that the algorithm combined with energy-aware transmission mechanism has a longer network lifetime as expected. Because of the imbalance consumption of energy near the sink, the uniform distribution has the shortest network lifetime. The nonuniform distribution combined with *T*_*t*_ achieved a shorter network lifetime than with *E*_*t*_, because its average energy consumption is larger than in *E*_*t*_ scheme. Our algorithm can achieve the optimal energy consumption among the whole working nodes, thus having the longest network lifetime.

## 6. Conclusion and Future Work

In this paper, we have investigated the problem of sensor redeployment to achieve optimal energy depletion and minimize sensor movement. We have given a theoretical analysis on energy consumption using nonuniform distribution strategy. Formally, we have proved that, the optimal energy consumption can be achieved through calculating the node densities for different regions of the target area. As a contribution, we have proposed an autonomous coverage-driven sensor redeployment algorithm to produce an optimal solution, which can maximize the network lifetime and minimize total movement of sensors. In addition, extensive simulation results have been presented to demonstrate the effectiveness of our proposed techniques.It also should be noted that we only consider the two dimensional (2D) case in this paper. As part of our future work, we will design new algorithms for the sensor redeployment problem in 3D space.

## Figures and Tables

**Algorithm 1:**
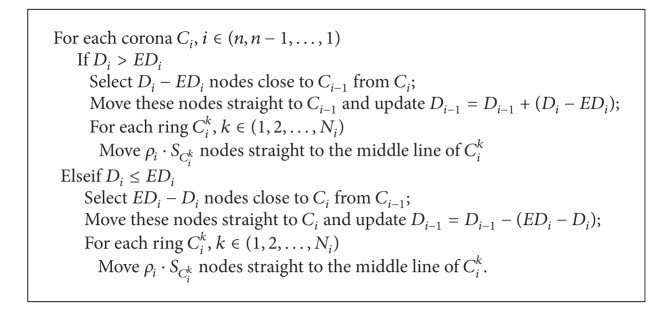


**Algorithm 2:**
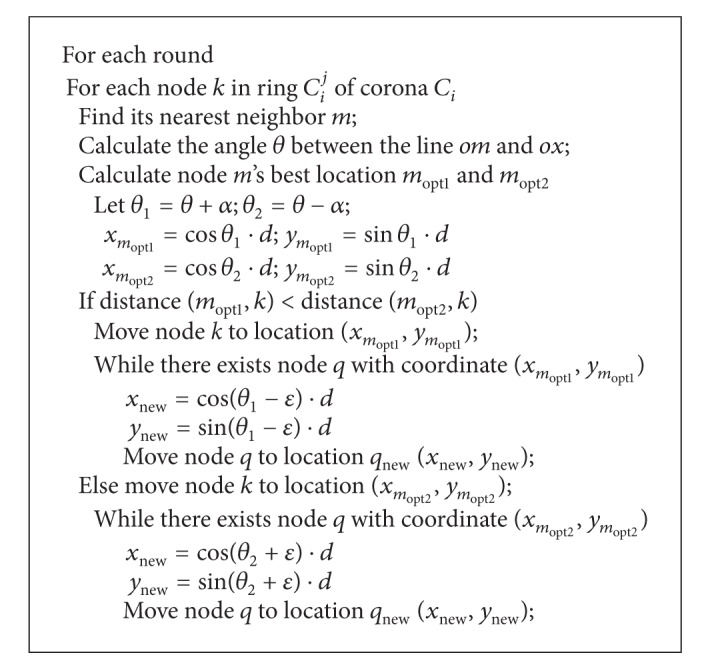


**Table 1:** Comparison of the number of working rounds.

Algorithm	The number of working rounds
Uniform deployment with *T*_*t*_	3
Uniform deployment with *E*_*t*_	17
Nonuniform deployment with *T*_*t*_	33
Nonuniform deployment with *E*_*t*_	61
Our proposed algorithm	104
